# Impact of Acute Pancreatitis Aetiology on Long-Term Outcomes Following a First Episode of Acute Pancreatitis: A Systematic Review and Meta-Analysis

**DOI:** 10.3390/jcm15093388

**Published:** 2026-04-29

**Authors:** Emmanuel Malesela Ndaba, Jones A. O. Omoshoro-Jones, Ekene Emmanuel Nweke, Pascaline N. Fru

**Affiliations:** 1 Department of Surgery, School of Clinical Medicine, Faculty of Health Sciences, University of the Witwatersrand, Johannesburg 2193, South Africa; omsjon@dr.com (J.A.O.O.-J.); ekene.nweke@wits.ac.za (E.E.N.); pascaline.fru@wits.ac.za (P.N.F.); 2 General & Hepatopancreatobiliary Surgery, Chris Hani Baragwanath Academic Hospital, Johannesburg 1864, South Africa

**Keywords:** acute pancreatitis, aetiology, chronic pancreatitis, exocrine pancreatic insufficiency, new-onset diabetes, long-term outcomes, systematic review, meta-analysis

## Abstract

**Background**: Acute pancreatitis (AP) is increasingly recognised as a disease with clinically significant long-term consequences. However, the extent to which aetiology influences the spectrum of long-term pancreatic sequelae remains unclear. This systematic review and meta-analysis evaluated long-term complications following a first episode of AP, with a protocol-defined focus on the impact of aetiology. **Methods**: This review evaluated eligible studies that included adults with a first episode of AP who were followed for chronic pancreatitis (CP), exocrine pancreatic insufficiency (EPI), or new-onset diabetes mellitus (NODM). A comprehensive search of PubMed, Scopus, Web of Science, and CENTRAL was conducted from January 2002 to June 2025. Risk of bias was assessed using the Newcastle–Ottawa Scale. Random-effects meta-analyses were performed to estimate pooled incidence proportions. A prespecified network meta-analysis was not feasible because outcome-specific event counts stratified by aetiology were inconsistently reported. This study satisfied PRISMA 2020 guidelines and was registered with PROSPERO (CRD420251074032). **Results**: Eight studies met eligibility criteria with extractable data for quantitative synthesis. Five studies (*n* ≈ 10,780) reported chronic pancreatitis (CP), with a pooled incidence of approximately 7–8% following a first episode of acute pancreatitis (AP) and substantial heterogeneity (*I*^2^ ≈ 96%). Three studies (*n* = 796) reported exocrine pancreatic insufficiency (EPI), with a pooled incidence of approximately 23%, although estimates were highly heterogeneous (*I*^2^ ≈ 98%). Four studies (*n* = 2706; 415 events) reported new-onset diabetes mellitus (NODM), with a pooled incidence of approximately 20% (*I*^2^ ≈ 93%). Although aetiology-specific quantitative comparisons were not possible, narrative synthesis consistently demonstrated higher long-term risk following alcohol-associated AP, lower risk after biliary AP, and intermediate but variable outcomes in idiopathic AP. **Conclusions**: Clinically meaningful long-term pancreatic dysfunction is common after a first episode of acute pancreatitis, particularly new-onset diabetes mellitus. While aetiology-specific risks could not be quantified, consistent patterns suggest that aetiology shapes long-term outcomes. These findings support structured, aetiology-informed follow-up after acute pancreatitis and the need for standardised outcome reporting in future studies.

## 1. Background

Acute pancreatitis (AP) is one of the most common gastrointestinal emergencies worldwide, with a rising incidence reported in both high-income and low-income regions [1,2]. Although historically regarded as a self-limiting inflammatory condition, increasing evidence indicates that AP may initiate a cascade of long-term structural and functional pancreatic changes. These include progression to chronic pancreatitis (CP), development of exocrine pancreatic insufficiency (EPI), and new-onset diabetes mellitus after pancreatitis (NODM), all of which carry substantial implications for quality of life, nutritional status, and long-term metabolic health [3,4].

Aetiology plays a pivotal role in determining the clinical course of AP and the likelihood of recurrence, severity, and long-term sequelae. Globally, gallstone-related and alcohol-associated AP account for the majority of cases, while hypertriglyceridaemia, post-procedural pancreatitis, medications, and idiopathic disease represent important secondary causes [5]. These aetiologies differ not only in their initiating mechanisms but also in their patterns of pancreatic injury and inflammatory burden. Comparative clinical studies have demonstrated that alcohol-associated AP is more likely to follow a recurrent and progressive course, whereas biliary AP is often a single-event disease with lower long-term risk once the obstructing gallstone has been removed [6,7]. Hypertriglyceridaemia-induced AP represents a distinct metabolic subtype and has been associated with greater disease severity, higher complication rates, and increased risk of recurrence, particularly in the absence of effective lipid control [8]. Despite growing recognition of these differences, heterogeneity in study design, outcome definitions, and follow-up duration has limited robust comparison of long-term outcomes across aetiologies.

Long-term outcomes following AP are increasingly recognised as clinically significant. Prospective cohorts and systematic reviews have shown that a proportion of patients experience recurrent acute pancreatitis and may progress to CP over time, even after a first episode [9,10]. Exocrine pancreatic insufficiency is also increasingly identified after AP, including among patients without established CP, with pooled analyses demonstrating a measurable burden of persistent or late-onset EPI following recovery from the index event [11,12]. In parallel, new-onset diabetes after pancreatitis has emerged as a common metabolic complication, with meta-analyses indicating that dysglycaemia may develop in a substantial subset of patients during long-term follow-up [13,14]. Collectively, these findings challenge the traditional view of AP as an isolated event and support a paradigm in which AP is recognised as a disease with potential chronic morbidity, underscoring the importance of structured, aetiology-informed long-term surveillance.

Despite several cohort studies addressing these outcomes, no comprehensive systematic review has synthesised the impact of AP aetiology on long-term pancreatic outcomes after a first episode of AP. Prior reviews have typically focused on recurrence, severity, or general prognosis, but not on the comparative influence of different aetiologies on CP, EPI, and NODM specifically. Furthermore, variations in study design, diagnostic criteria, and time points make it challenging to draw definitive conclusions from individual studies.

This systematic review and network meta-analysis was therefore designed to evaluate the impact of AP aetiology on long-term outcomes, including chronic pancreatitis, exocrine pancreatic insufficiency, and new-onset diabetes. Although quantitative comparative modelling between aetiologies was limited by inconsistent reporting in the available literature, this review integrates both aetiology-focused narrative synthesis and pooled incidence estimates to provide a comprehensive overview of long-term pancreatic morbidity following a first episode of AP. The findings aim to inform personalised follow-up strategies and highlight key areas for future research.

## 2. Methods

This systematic review and planned network meta-analysis were conducted in accordance with the Preferred Reporting Items for Systematic Reviews and Meta-Analyses (PRISMA) 2020 guidelines. The review protocol was prospectively registered with the International Prospective Register of Systematic Reviews (PROSPERO; registration number CRD420251074032). The completed PRISMA 2020 checklist is provided in the Supplementary Materials. Ethical approval was waived by the Human Research Ethics Committee of the University of the Witwatersrand (reference number R14/49), as this study involved analysis of previously published data only.

The primary objective, as prespecified in the registered protocol, was to evaluate the impact of acute pancreatitis (AP) aetiology on long-term pancreatic outcomes following a first episode of AP. All methodological steps were performed in accordance with the registered protocol unless explicitly modified due to limitations in the available data; any such deviations are described and justified below.

### 2.1. Eligibility: Criteria

Eligible studies included prospective or retrospective cohort studies, population-based cohorts, and cross-sectional studies with longitudinal follow-up reporting long-term pancreatic outcomes after a first episode of AP. Studies were required to include adults aged 18 years or older, with AP diagnosed based on at least two recognised criteria: characteristic pancreatic-type abdominal pain, serum amylase or lipase ≥ 3 times the upper limit of normal, or imaging features consistent with AP. Only studies that clearly identified a first episode of AP and reported at least one protocol-defined long-term outcome were included. Excluded were case reports, small case series (<10 participants), reviews, systematic reviews and meta-analyses, conference abstracts without extractable data, interventional trials not reporting long-term outcomes, animal studies, and studies combining first-episode AP with recurrent AP without separable data.

### 2.2. Outcomes

Three primary long-term outcomes were prespecified:

1. Chronic pancreatitis (CP), defined by clinical, imaging, or validated diagnostic criteria.
2. Exocrine pancreatic insufficiency (EPI), defined by fecal elastase-1 < 200 µg/g, abnormal 72-h fecal fat, or consistent clinical evidence.
3. New-onset diabetes mellitus (NODM), defined using WHO or ADA diagnostic criteria or validated registry definitions [15,16,17].

Aetiological categories were extracted whenever reported, including alcohol, biliary, idiopathic, hypertriglyceridaemia, post-ERCP, and drug-induced AP. Although aetiology was consistently documented, outcome-specific event counts stratified by aetiology were incomplete across studies, limiting the feasibility of quantitative aetiology comparisons.

### 2.3. Information Sources and Search Strategy

A comprehensive search of PubMed/MEDLINE, Scopus, Web of Science Core Collection, and the Cochrane CENTRAL database was conducted from January 2002 to June 2025. Search strategies combined controlled vocabulary and free-text terms relating to acute pancreatitis, aetiology, and long-term pancreatic outcomes. Manual reference list screening, citation tracking, and targeted grey literature searches were also performed to maximise study capture.

### 2.4. Study Selection

**Figure 1:**
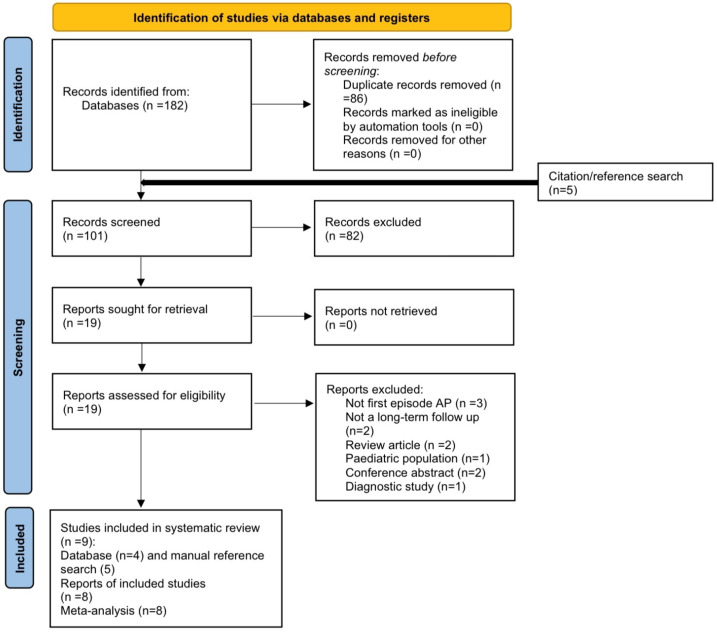
Flow diagram illustrating study identification, screening, eligibility assessment, and inclusion. The flow diagram was generated following the PRISMA 2020 guidelines [19].

All search results were imported into Rayyan (Version 1.7.2; Rayyan systems Inc., Cambridge, MA, North America, USA) for blinded, independent screening by three reviewers [18]. Duplicate records were identified and removed prior to screening. Titles and abstracts were then screened for eligibility, followed by full-text assessment of potentially relevant reports. Any discrepancies between reviewers were resolved by consensus. Reasons for exclusion at the full-text stage were documented. The study selection process is summarised using a PRISMA 2020 flow diagram (Figure 1) [19].

### 2.5. Data Extraction

Three reviewers independently extracted data using a piloted extraction form. Extracted variables included study characteristics (author, year, country, design), demographic information, AP severity, aetiology, definitions of CP, EPI, and NODM, follow-up duration, and extractable numerators and denominators for each outcome. All extracted values were cross-checked for accuracy. The study characteristics are outlined in Table 1.

### 2.6. Risk-of-Bias Assessment

Risk of bias in observational studies was assessed using the Newcastle–Ottawa Scale (NOS). Ref. [27] evaluates cohort selection, comparability, and outcome ascertainment. Studies were categorised as low (7–9 points), moderate (5–6 points), or high risk (<5 points), and results are presented in Table 2.

### 2.7. Certainty of Evidence Assessment

The certainty of evidence for each primary outcome was assessed using the Grading of Recommendations Assessment, Development and Evaluation (GRADE) approach [28]. A summary of the ratings is reported in Table 3.

### 2.8. Statistical Analysis

The protocol prespecified two analytical components: (1) a network meta-analysis (NMA) comparing long-term outcomes across acute pancreatitis aetiologies and (2) pooled incidence estimation for CP, EPI, and NODM.

However, none of the included studies provided consistent, outcome-specific numerators stratified by aetiology. In accordance with established methodological standards, the NMA was therefore not performed, and the influence of aetiology on long-term outcomes was synthesised narratively.

Quantitative pooling of the three primary outcomes was performed using Review Manager (RevMan) version 5.4 (Cochrane Collaboration). For each outcome, pooled incidence proportions were calculated using an inverse-variance random-effects model, accounting for between-study heterogeneity. Proportions were analysed on the logit scale to stabilise variances, particularly in studies with low or high event rates, and back-transformed to obtain pooled incidence estimates with corresponding 95% confidence intervals.

Individual study confidence intervals were calculated using exact binomial (Clopper–Pearson) methods, as reported in the original studies [29]. Statistical heterogeneity was assessed using Cochran’s Q test [30] and quantified using the I^2^ statistic [31], with higher values indicating increasing between-study variability.

Sensitivity analyses included visual inspection of forest plots and assessment of consistency in effect direction across studies. Formal assessment of publication bias was not undertaken because fewer than ten studies contributed data to each outcome, in accordance with Cochrane methodological guidance. All analyses were conducted in line with PRISMA 2020 recommendations [19].

## 3. Results

A total of 182 records were identified across electronic databases. After removal of duplicates, 96 titles and abstracts were screened, and 19 full-text articles were assessed for eligibility. Four studies met the inclusion criteria from the database search, and a further four were identified through manual reference list screening and citation tracking, resulting in eight studies included in the final review.

### 3.1. Study Characteristics

Eight observational cohort studies [9,20,21,22,23,24,25,26,32] met the inclusion criteria and were published between 2004 and 2025, comprising prospective and retrospective designs. These studies represented patient populations from Europe, Asia, and North America. Sample sizes ranged from 75 to 7456 participants, with follow-up durations varying from 12 months to more than 5 years. All included studies met the inclusion criteria. Severity spectra generally included mild, moderately severe, and severe AP, although definitions and follow-up assessment methods varied across studies.

Across the included studies, five [9,21,23,24,26] reported CP as a long-term outcome, three [22,23,26] reported EPI, and four [9,22,25,26] reported NODM. Several studies contributed data to more than one outcome domain. Definitions and diagnostic criteria for CP, EPI, and NODM varied substantially across studies, including differences in imaging modalities, functional testing strategies (including fecal elastase-1 thresholds), biochemical definitions, and follow-up intensity and duration.

Aetiological composition differed across cohorts but was broadly harmonised into four major categories: biliary (gallstone-related), alcohol-related, idiopathic, and other or composite causes. Pooled patient-level aetiology data were derived from six cohort studies [9,20,21,24,25,26], comprising 13,014 participants. Biliary acute pancreatitis accounted for the largest proportion of index events (35.8%), followed by idiopathic (23.9%), other or composite causes (23.6%), and alcohol-related acute pancreatitis (16.6%). Although alcohol-related acute pancreatitis is a well-established risk factor for recurrent and progressive pancreatic disease, its proportional representation in pooled patient-level analyses was lower than biliary and composite “other” causes, largely reflecting the dominance of large population-based cohorts, heterogeneous aetiology classification, and aggregation of multiple non-biliary, non-alcohol causes within the “other” category.

Outcome-specific event counts stratified by pancreatitis aetiology were inconsistently reported across studies, precluding the prespecified network meta-analysis. The available data did not meet minimum connectivity requirements for comparative modelling; therefore, aetiology-specific patterns were synthesised narratively.

Across studies, alcohol-related acute pancreatitis was more frequently associated with higher reported rates of chronic pancreatitis, exocrine pancreatic insufficiency, and new-onset diabetes mellitus. Biliary pancreatitis generally demonstrated lower progression rates across outcomes, whereas idiopathic pancreatitis showed intermediate patterns with substantial between-study variability. Hypertriglyceridaemia-associated and post-ERCP pancreatitis were inconsistently reported and lacked sufficient outcome-specific data for meaningful comparative assessment. The overall distribution of pancreatitis aetiology across the included cohorts is summarised in Table 4.

### 3.2. Chronic Pancreatitis

Five studies (*n* ≈ 10,780) contributed data on progression to chronic pancreatitis (CP). Reported incidence ranged from 3.7% to 13.5%, with a pooled estimate of approximately 7–8% following an episode of acute pancreatitis. This finding was statistically significant and indicates that progression to CP is a clinically relevant long-term outcome.

**Figure 2:**
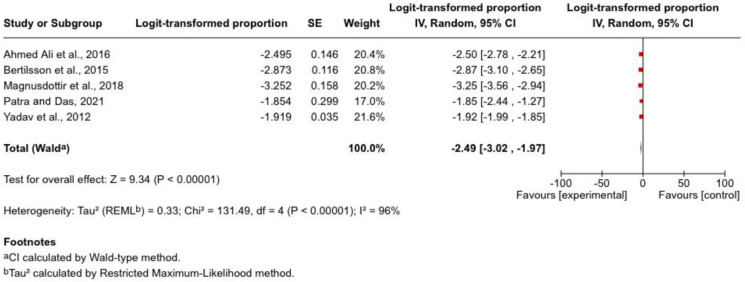
Forest plot of pooled incidence of chronic pancreatitis following acute pancreatitis [9,21,23,24,26]. Forest plot showing individual study estimates and pooled incidence of chronic pancreatitis following acute pancreatitis using a random-effects meta-analysis model. Squares represent study-specific estimates with corresponding 95% confidence intervals, and the diamond represents the pooled estimate.

Substantial heterogeneity was observed (*I*^2^ = 96%), likely reflecting differences in study design, follow-up duration, aetiology distribution, and diagnostic criteria. Studies with longer follow-up and population-based designs tended to report higher cumulative incidence, suggesting that CP risk increases over time. Higher progression rates were noted in cohorts with a greater proportion of alcohol-associated or severe pancreatitis; however, aetiology-specific data were insufficient for formal subgroup comparison. The pooled effect estimate and between-study variability for progression to chronic pancreatitis are illustrated in Figure 2.

### 3.3. Exocrine Pancreatic Insufficiency

Three studies (*n* = 796) provided data on exocrine pancreatic insufficiency (EPI) following acute pancreatitis. Reported incidence varied widely, ranging from 5.1% in a large population-based cohort to over 50% in a study using direct pancreatic function testing. The pooled estimate suggested an approximate incidence of 23%; however, this was not statistically significant.

**Figure 3:**
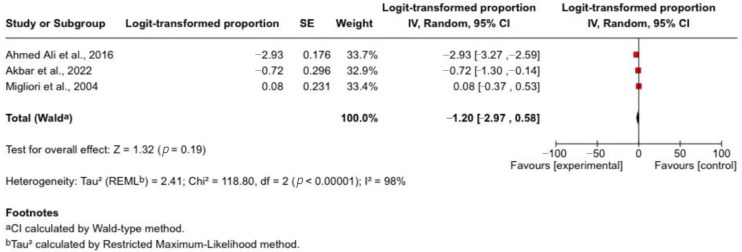
Forest plot of pooled incidence of exocrine pancreatic insufficiency following acute pancreatitis [22,23,26]. Forest plot illustrating the pooled incidence of exocrine pancreatic insufficiency after acute pancreatitis. A random-effects model was used to account for between-study heterogeneity. Horizontal lines indicate 95% confidence intervals.

There was substantial heterogeneity across studies (*I*^2^ = 98%), likely reflecting differences in disease severity, diagnostic methods (faecal elastase-1 versus direct function testing), and timing of assessment. These findings indicate that while EPI is a clinically relevant complication after acute pancreatitis, its reported incidence varies considerably depending on diagnostic approach and patient selection. Accordingly, pooled estimates should be interpreted with caution. Three individual study estimates and the pooled incidence of exocrine pancreatic insufficiency following acute pancreatitis are shown in Figure 3.

### 3.4. New-Onset Diabetes Mellitus

Four studies (*n* = 2706; 415 events) reported new-onset diabetes mellitus (NODM) following acute pancreatitis. Reported incidence ranged from approximately 13% to 20%. The pooled estimate suggested an incidence of approximately 20%, indicating that NODM is a common long-term metabolic complication after acute pancreatitis. Time-to-event data from longitudinal cohorts indicate that new-onset diabetes may occur within months after the index episode of acute pancreatitis and continues to accumulate over subsequent years. In a large population-based cohort, the median time to diabetes onset was 440 days among critically ill patients and 1115 days among those managed on general wards. Prospective data also demonstrated a progressive increase in endocrine dysfunction within the first year, with diabetes identified in 1.16% at 3 months, 5.81% at 6 months, and 10.5% at 12 months.

**Figure 4:**
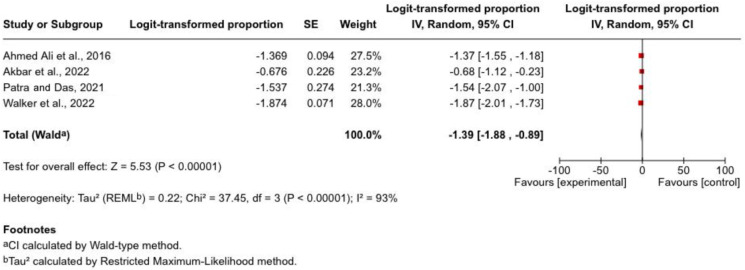
Forest plot of pooled incidence of new-onset diabetes mellitus following acute pancreatitis [9,22,25,26]. Forest plot depicting the pooled incidence of new-onset diabetes mellitus (NODM) following acute pancreatitis, defined according to WHO or ADA diagnostic criteria. Study-level estimates and the pooled effect are presented with 95% confidence intervals.

Substantial heterogeneity was observed (*I*^2^ = 93%), likely reflecting differences in follow-up duration, diagnostic criteria, and study populations. Although variability existed, all included studies demonstrated a clinically meaningful burden of post-pancreatitis diabetes. Shorter-duration cohorts tended to report higher early NODM rates, whereas studies with longer follow-up showed more stable incidence estimates, suggesting possible partial endocrine recovery over time. The pooled analysis is illustrated in Figure 4.

## 4. Sensitivity Analyses

Sensitivity analyses demonstrated that fixed-effects models yielded point estimates consistent with the primary inverse-variance random-effects analyses, with narrower confidence intervals, as expected. Leave-one-out influence analyses confirmed that no individual study materially altered the pooled estimates for any outcome. The direction and magnitude of effects remained stable across sensitivity analyses, supporting the robustness of the findings despite substantial between-study heterogeneity.

Owing to the inclusion of fewer than ten studies per outcome, formal assessment of publication bias (e.g., funnel plots or regression-based asymmetry tests) was not undertaken.

## 5. Discussion

This systematic review and meta-analysis examined long-term pancreatic outcomes following a first episode of AP, with particular interest in the role of aetiology in shaping chronic disease trajectories. Across eight observational studies, clinically relevant incidences of CP, EPI, and NODM were identified, reinforcing the concept that even a single episode of AP may initiate lasting structural and functional pancreatic impairment. These findings highlight the long-term burden of post-pancreatitis sequelae and provide a framework for interpreting heterogeneity across study designs and populations.

Among the evaluated outcomes, progression to CP represented the most robustly characterised endpoint, supported by the largest number of contributing studies and participants.

Despite substantial between-study heterogeneity, the direction of effect for CP progression was consistent across studies, indicating that a meaningful subset of patients progress to chronic pancreatic disease even after a single episode of acute pancreatitis [9,21,23,24,26]. These findings align with existing evidence suggesting that acute pancreatitis may act as an initiating event within a continuum of pancreatic injury rather than an isolated, self-limited episode [10].

Chronic pancreatitis (CP) represents a heterogeneous disease entity and is not solely the consequence of relapsing acute pancreatitis. While recurrent acute inflammatory episodes may contribute to progressive fibrosis in some patients, CP may also arise independently due to alcohol-related injury, genetic susceptibility, autoimmune mechanisms, or idiopathic processes [33]. Furthermore, an episode of acute pancreatitis may accelerate the natural history of pre-existing or subclinical CP, thereby complicating causal interpretation.

Endocrine dysfunction emerged as a clinically important long-term outcome following acute pancreatitis. Prior meta-analyses have demonstrated that new-onset diabetes mellitus is a common complication after AP, challenging the paradigm of AP as a metabolically reversible condition [3,13]. By restricting inclusion to first-episode AP and evaluating endocrine dysfunction alongside structural and exocrine outcomes, the present analysis extends these findings, with pooled estimates indicating that approximately 16–20% of patients develop NODM after an initial episode. This supports the interpretation that endocrine dysfunction is not solely attributable to recurrent or severe disease but represents a frequent long-term sequela after a first AP episode as well [3,13].

The duration of follow-up is critical when interpreting the incidence of post-pancreatitis diabetes, as risk appears to evolve dynamically over time [22,25].

Therefore, studies with limited follow-up may underestimate true incidence, and longitudinal surveillance extending beyond the early post-discharge period is necessary to fully characterize risk.

In comparison with the general population, where global age-standardised diabetes prevalence is approximately 6% and annual incidence estimates range from 0.5–1% per year [34], the pooled incidence of new-onset diabetes following acute pancreatitis approached 20%. Even accounting for differences in follow-up duration, this suggests a substantially elevated metabolic risk after a first episode of acute pancreatitis, supporting the concept of post-pancreatitis diabetes as a distinct high-risk state.

EPI demonstrated the greatest uncertainty among the evaluated long-term outcomes. Previous systematic reviews have shown that EPI is common during admission for AP and persists in a substantial proportion of patients during follow-up, albeit with marked heterogeneity driven by differences in disease severity, diagnostic modalities, and timing of assessment [11]. The findings of this review are concordant with this variability, characterised by wide confidence intervals and high heterogeneity. Importantly, by placing EPI alongside CP and NODM within a unified analytical framework, the present study highlights that inconsistency in reported EPI incidence likely reflects methodological heterogeneity and under-recognition rather than a negligible disease burden [11].

Collectively, existing literature supports the concept that acute pancreatitis frequently results in long-term pancreatic dysfunction; however, prior studies have often evaluated outcomes in isolation or within heterogeneous populations that included recurrent or mixed AP cohorts [10,11,13]. By focusing on first-episode acute pancreatitis, simultaneously assessing chronic, exocrine, and endocrine sequelae, and applying contemporary random-effects meta-analytic methods, the present study refines and extends existing knowledge. These findings further challenge the view of acute pancreatitis as a benign, self-limited condition and underscore the need for structured long-term surveillance long after a single episode of acute pancreatitis [3,10,11,13].

### 5.1. Role of Aetiology in Long-Term Outcomes After Acute Pancreatitis

Aetiology appears to function as a key biological modifier of long-term pancreatic outcomes following AP, rather than acting solely as a confounding variable [10]. Across the included studies, alcohol-related AP was consistently associated with higher rates of progression to CP, as well as increased risks of EPI and NODM, compared with biliary or idiopathic causes [9,20,21,22,23,24,25,26,32]. This pattern was observed across multiple study designs, including large population-based cohorts, suggesting a reproducible aetiology-specific signal rather than isolated findings [9,20,21,22,23,24,25,26,32].

Alcohol-associated AP demonstrated the highest progression rates to CP in several cohorts, even after accounting for disease severity and recurrence. In population-based and observational studies, alcohol aetiology remained independently associated with CP development, supporting the concept of sustained pancreatic injury driven by ongoing toxic exposure and impaired regenerative capacity [35,36]. These findings align with established biological plausibility, whereby continued alcohol consumption may perpetuate inflammation, fibrosis, and ductal injury after resolution of the index AP episode [9,21,23,24,26].

Although our findings demonstrate an increased risk of endocrine and exocrine dysfunction following alcoholic acute pancreatitis, alcohol exposure itself is independently associated with chronic pancreatitis and pancreatic metabolic impairment [37]. Therefore, in patients with alcoholic etiology, subsequent dysfunction may reflect both the inflammatory impact of the index acute episode and underlying alcohol-related pancreatic injury. Accordingly, etiology-specific risk estimates should be interpreted with consideration of alcohol as a potential confounding and disease-modifying factor.

In contrast, biliary AP was consistently associated with lower long-term progression rates across CP, EPI, and NODM outcomes [9,20,21,22,23,24,25,26,32]. This likely reflects the reversible nature of the inciting insult, as gallstone-related obstruction is often definitively treated through cholecystectomy or endoscopic intervention, thereby limiting recurrent pancreatic injury [38]. These data support the concept that timely removal of the causative factor may mitigate long-term pancreatic damage following AP [39].

Idiopathic AP demonstrated intermediate and highly variable long-term outcomes, a finding that likely reflects underlying heterogeneity within this diagnostic category [9,20,21,24,26,32]. Misclassification of occult biliary disease, unrecognised alcohol exposure, metabolic factors, or genetic predisposition may contribute to this variability [38]. As such, idiopathic AP should not be assumed to carry a uniformly benign prognosis, particularly in the absence of thorough aetiological evaluation.

### 5.2. Severity, Necrosis and Disease Progression

Severity-related factors appear to influence long-term outcomes after a first episode of acute pancreatitis. Greater exocrine dysfunction has been reported in patients with alcoholic and necrotising disease, with persistence at follow-up [23]. In population-based cohorts with extended follow-up of approximately 4–5 years, local complications, organ failure, and recurrent acute pancreatitis were independently associated with progression to chronic pancreatitis [20,24]. Peripancreatic necrosis was also linked to increased recurrence risk in these cohorts [24]. In a shorter prospective follow-up (12 months), endocrine dysfunction increased over time and was independently associated with intervention for walled-off necrosis, suggesting a relationship between necrotic disease burden and metabolic sequelae [22].

However, variability in follow-up duration and differences in severity classification across studies limit direct comparison and preclude precise quantification of severity as an independent predictor in pooled analyses. Severity and necrosis were inconsistently reported across studies; therefore, severity could not be formally assessed as an independent predictor, representing a limitation of this meta-analysis.

Although time-to-event data highlight that diabetes may occur within months and continues to accumulate over several years after acute pancreatitis, the available literature does not consistently report person-time incidence (e.g., cases per 1000 person-years) or provide uniform adjustment for recurrent pancreatitis and incident chronic pancreatitis across cohorts. Therefore, we were unable to derive a pooled annualized incidence rate or perform meta-analytic correction for relapse and chronic pancreatitis, and this remains an important limitation of the current evidence base.

### 5.3. Local Complications and Long-Term Outcomes

Local pancreatic complications are common after acute pancreatitis (AP). In a 5-year longitudinal cohort of patients with first-episode AP, acute fluid collections were present in 83% of patients during the index admission, and 46% of these progressed to encapsulated late collections (pseudocyst or walled-off necrosis (WON)) [9]. Previous cohort studies have also reported the occurrence of pancreatic pseudocysts following acute pancreatitis; for example, Maringhini et al. described an incidence of approximately 5% in patients with non-alcoholic acute pancreatitis [40]. At 5-year follow-up, 13% of patients had developed chronic pancreatitis. Although pseudocyst/WON was more frequent among patients who later developed recurrent AP or chronic pancreatitis, this association was not statistically significant. In contrast, overall disease severity at the index episode was associated with long-term progression, suggesting that the extent of pancreatic injury rather than the mere presence of a pseudocyst may influence structural outcomes [9].

In a separate prospective study focusing on metabolic sequelae, patients requiring intervention for WON had a markedly increased risk of developing new-onset endocrine dysfunction within 12 months [22]. Taken together, these findings indicate that while pseudocysts are frequent after AP, long-term progression to chronic pancreatitis and diabetes appears to be more closely related to necrotizing disease and overall severity than to fluid collections alone.

### 5.4. Strengths and Limitations

#### 5.4.1. Strengths

This study has several important strengths. Notably, it focuses specifically on long-term pancreatic outcomes following a first episode of acute pancreatitis, thereby isolating disease trajectories that arise after an initial inflammatory insult rather than those driven by recurrent attacks or established chronic disease. Secondly, the study simultaneously evaluated structural (chronic pancreatitis), exocrine (exocrine pancreatic insufficiency), and endocrine (new-onset diabetes mellitus) outcomes within a single analytical framework, allowing a comprehensive assessment of protracted pancreatic dysfunction.

Third, methodological robustness was supported by the use of random-effects models, sensitivity and influence analyses, and alternate statistical transformations, which demonstrated that no single study disproportionately influenced the pooled estimates.

#### 5.4.2. Limitations

Several limitations warrant consideration. Most notably, substantial between-study heterogeneity was observed across all outcomes, reflecting differences in follow-up duration, patient populations, acute pancreatitis severity, and study design. While heterogeneity limits the precision of pooled incidence estimates, it likely reflects genuine clinical variability rather than methodological error and underscores the complexity of ongoing pancreatic disease evolution.

Second, diagnostic inconsistency across studies represents a key limitation, particularly for exocrine pancreatic insufficiency and chronic pancreatitis. Definitions of chronic pancreatitis ranged from imaging-based criteria to administrative coding, while EPI diagnosis varied widely in terms of testing modality, thresholds, and timing of assessment. This variability likely contributed to the wide confidence intervals observed and may have resulted in underestimation or misclassification of true disease burden.

Third, aetiology-specific quantitative analyses could not be performed, as most studies did not report outcome-specific numerators stratified by aetiology. Although narrative synthesis consistently suggested higher progression rates in alcohol-related acute pancreatitis and lower rates in biliary disease, the lack of harmonised reporting precluded formal comparative modelling.

Finally, the relatively small number of studies contributing to each outcome limited the ability to formally assess publication bias and explore subgroup effects through meta-regression. Nonetheless, influence analyses indicated that pooled estimates were not driven by individual studies.

## 6. Conclusions

This systematic review and meta-analysis demonstrate that lasting pancreatic sequelae are common following a first episode of acute pancreatitis and challenge the traditional view of acute pancreatitis as a fully reversible condition.

Furthermore, our findings support the concept of aetiology as a biological modifier of disease trajectory rather than a simple confounder and highlight the need for harmonised, outcome-specific reporting in future studies.

Collectively, these results have important clinical implications. Patients recovering from a first episode of acute pancreatitis may benefit from structured long-standing surveillance, particularly those with severe disease or alcohol-associated aetiology. Future prospective studies with standardised definitions, aetiology-stratified outcomes, and comprehensive assessment of both exocrine and endocrine function are essential to refine risk stratification and inform evidence-based follow-up strategies.

## Figures and Tables

**Table 1:** Characteristics of cohort studies evaluated for long-term outcomes following first-episode acute pancreatitis.

Study (Year)	Country	Study Design	Population (N)	Mean Age	Males	First AP Only	Follow-Up Duration	Chronic Pancreatitis (n/%)	Exocrine Pancreatic Insufficiency (EPI)	New-Onset Diabetes (NODM)	Outcomes Usable for Meta-Analysis	Aetiology of AP
Magnusdottir et al., 2018 [20]	Iceland	Prospective cohort	1102	56	54%	Yes	Mean 4 years	41 (3.7%)	NR	NR	CP	Biliary: 41% (452), Alcoholic 21% (231) Idiopathic 26% (287) Other causes 13% (143)
Yadav et al., 2012 [21]	USA	Retrospective cohort	7 456	58 +/- 20 years	45%	Yes	Median 40 months (IQR 18–69)	1401 (18.6%)	NR	NR	CP	Biliary; 28% (2088) Alcohol; 19% (1417) Idiopathic; 36% (2684) Other causes; 17% (1267)
Akbar et al., 2022 [22]	India	Prospective cohort	86	33.0 years (IQR 26.0–44.2)	77%	Yes	6,and 12 months	NR	NRI	29/86 33.7%	NODM	Biliary; 17 (19.8%) Alcohol 31 (36%) Other
Migliori et al., 2004 [23]	Italy	Prospective cohort	75	45.9 years (range 17–80)	(76%)	Mixed	Long-term	NR	39 (52.0%)	NR	EPI	Biliary: 52% Alcoholic AP: 48%
Bertilsson et al., 2015 [24]	Sweden	Population-based retrospective cohort	1457	61	53%	Yes	Median 4.6 years	78/1457 (5.4%)	NR	NR	CP	Biliary: 704 (48%) Alcohol-associated: 249 (17%) Other known: 72 (5%) Idiopathic: 432 (30%)
Patra & Das, 2021 [9]	India	Retrospective cross-sectional	96	42 years (range 14–88 years)	64%	Yes	5 years	13/96 (13.5%)	NR	17/96 (17.7%)	CP, NODM	Alcohol-related: ≈52% Biliary (gallstones): ≈24% Idiopathic: ≈15% Other causes (hypertriglyceridaemia, drugs, post-ERCP, etc.): ≈9%
Walker et al., 2022 [25]	Scotland	Nationwide EHR-linked cohort	2047	56 years	49.8%	Yes	≥5 years	NR	NR	DM 232/1748 (13.3%)	NODM	Gallstone-related AP: 958/2047 (46.8%) Non-gallstone AP (composite: alcohol + other): 1089/2047 (53.2%)
Ahmed Ali et al., 2016 [26]	Netherlands	Multicentre observational cohort	669	57 years (IQR 42–70)	55%	Yes	Median 57 months	51/669 (7.6%)	34 (5.1%)	136 1(20%)	CP, EPI, NODM	Biliary 384 (58%) Alcohol 153 (23%) Idiopathic 108 (15%) Other 24 (4%)

**Table 2:** Risk-of-bias assessment of included cohort studies using the Newcastle–Ottawa Scale (NOS).

Study	Selection (0–4)	Comparability (0–2)	Outcome (0–3)	Total (0–9)	Overall Risk
Magnusdottir et al., 2018 [20]	4	2	3	9	Low
Yadav et al., 2012 [21]	4	2	2	8	Low
Akbar et al., 2022 [22]	3	1	2	6	Moderate
Migliori et al., 2004 [23]	3	1	2	6	Moderate
Bertilsson et al., 2015 [24]	4	2	2	8	Low
Patra & Das, 2021 [9]	3	1	2	6	Moderate
Walker et al., 2022 [25]	4	2	3	9	Low
Ahmed Ali et al., 2016 [26]	4	2	2	8	Low

Abbreviations: NOS, Newcastle–Ottawa Scale. Selection (0–4), comparability (0–2), and outcome (0–3) domains represent the standard Newcastle–Ottawa Scale scoring system for cohort studies, with total scores ranging from 0 to 9. Overall risk of bias was classified as low (7–9), moderate (5–6), or high (≤4).

**Table 3:** GRADE summary of findings for long-term pancreatic outcomes following a first episode of acute pancreatitis.

Outcome	No. of Studies (Participants)	Pooled Incidence (Approx.)	Risk of Bias	Inconsistency	Indirectness	Imprecision	Publication Bias	Overall Certainty (GRADE)
Chronic pancreatitis (CP)	5 (~10,780)	~7–8%	Serious (observational cohorts; variable follow-up/ascertainment)	Very serious (*I*^2^ ~96%)	Serious (variable CP definitions/diagnostic pathways)	Not serious–Serious (precision acceptable overall, but variability across studies)	Undetected/Not assessed (<10 studies)	Very low
Exocrine pancreatic insufficiency (EPI)	3 (~796)	~23% (not statistically significant overall)	Serious (ascertainment + selection differences)	Very serious (*I*^2^ ~98%)	Very serious (different diagnostic tests; timing-dependent detection)	Serious (wide uncertainty + small evidence base)	Undetected/Not assessed (<10 studies)	Very low
New-onset diabetes mellitus (NODM)	4 (2706; 415 events)	~20%	Serious (observational; diabetes ascertainment may vary)	Very serious (*I*^2^ ~93%)	Serious (variable diagnostic criteria and follow-up timepoints)	Not serious–Serious (moderate precision but heterogeneity high)	Undetected/Not assessed (<10 studies)	Very low

**Table 4:** Pooled aetiology distribution.

Aetiology	*n*	%
Biliary	4665	35.8%
Alcohol-related	2167	16.6%
Idiopathic	3108	23.9%
Other/composite	3074	23.6%
Total	13,014	100%

Pooled patient-level aetiology data were available for 13,014 patients. Biliary aetiology was the most common (35.8%), followed by idiopathic (23.9%), other causes (23.6%), and alcohol-related acute pancreatitis (16.6%).

## Data Availability

The data supporting the findings of this study are available within the published literature indexed in PubMed/MEDLINE, Scopus, Web of Science Core Collection, and the Cochrane CENTRAL database. Extracted data are presented within the article and its Supplementary Materials.

## Supplementary Materials

The following supporting information can be downloaded at: https://www.mdpi.com/article/10.3390/jcm15093388/s1, Table S1: The PRISMA 2020 checklist.

## Abbreviations

AP: Acute pancreatitis; CP: Chronic pancreatitis; EPI: Exocrine pancreatic insufficiency; NODM: New-onset diabetes mellitus; IAP: Idiopathic acute pancreatitis; ERCP: Endoscopic retrograde cholangiopancreatography; EUS: Endoscopic ultrasound; CT: Computed tomography; MRI: Magnetic resonance imaging; MRCP: Magnetic resonance cholangiopancreatography; NOS: Newcastle–Ottawa Scale; GRADE: Grading of Recommendations Assessment, Development and Evaluation
